# Case Report: Successful Outcome for Refractory Diabetic Peripheral Neuropathy in Patients With Ultrasound-Guided Injection Treatment

**DOI:** 10.3389/fendo.2021.735132

**Published:** 2021-10-28

**Authors:** Hua Qiong Hu, Hailun Huang, Jing Huang, Ji Cui Leng, Mi Li, Chao Tang, Yan Li, Shan Wu

**Affiliations:** Department of Neurology, Affiliated Hospital of Guizhou Medical University, Guiyang, China

**Keywords:** hydrodissection, peripheral nerve compression, the scoring system of ultrasound, electrophysiological, refractory diabetic peripheral neuropathy

## Abstract

Diabetic peripheral neuropathy is the most prevalent chronic complication of diabetes and is based on sensory and autonomic nerve symptoms. Generally, intensive glucose control and nerve nourishment are the main treatments. However, it is difficult to improve the symptoms for some patients; such cases are defined as refractory diabetic peripheral neuropathy (RDPN). In this paper, we present five patients treated with saline and mecobalamin by ultrasound-guided injection. The Visual Analog Scale and Toronto Clinical Scoring System were used to evaluate the symptoms, and the neuro-ultrasound scoring system and electrophysiological severity scale were evaluated by ultrasound and electrophysiological examination. In brief, ultrasound-guided hydrodissection may be a safe way to treat RDPN.

## Introduction

Hydrodissection was first proposed in 1997. It is used in treating entrapment neuropathy and inflammatory peripheral neuropathy and plays an important role in separating adjacent tissue and protecting nerves and blood vessels *via* the injection of saline or other agents to build a non-existent surgical plane (1, 2). Previously, Smith has studied the treatment of carpal tunnel syndrome by hydrodissection (3).Without a unified standard, a visual needle is usually used to form a liquid clearance around nerves by ultrasound-guided injection with saline or another liquid (e.g., mecobalamin) to dissociate the nerves from their surroundings.

Currently, the treatment options for compressive neuropathy include: (1) medical treatment with oral drugs to provide nutrition to the nerves and blocking therapy (4) and (2) surgical treatment, after which symptoms may recur. Surgical procedures may damage the intraneural microcirculation, leading to pathological changes corresponding to chronic compressive neuropathy (5). However, hydrodissection minimizes the risk of nerve injury with its advantages of percutaneous punctures and blunt dissection. Ultrasound shows changes in the morphology, imaging, and echo of nerves, which could suddenly be attenuated at compressive sites, resulting in decreased echo intensity. In fact, honeycomb-like structures disappear on the axis, and the cross-sectional areas of proximal nerves increase due to edema. Therefore, ultrasound-guided neural hydrodissection represents a promising therapeutic strategy to treat various peripheral neuropathies.

Over the past decades, various investigations have been performed on neuropathies with mild to moderate, recurrent, and even severe nerve scars to explore the therapeutic effects of hydrodissection (6–9).

Hydrodissection has therapeutic effects on neural adhesion caused by inflammation, such as saphenous nerve ache. Fader has cured an athlete with compression of the right sural nerve on the right lateral foot and ankle using saline by hydrodissection to separate the sural nerve from the surrounding muscles; the athlete returned to participation in competitive sports without disability (10).

Based on 10 years of clinical experience, in this study, we demonstrate that ultrasound-guided neural hydrodissection has excellent therapeutic effects on compressive neuropathy and inflammatory adhesion and explore the therapeutic efficacy of ultrasound-guided injection in five patients with refractory peripheral neuropathy.

## Patients and Methods

Five patients treated with intramuscular or oral mecobalamin were recruited. They were diagnosed with refractory diabetic peripheral neuropathy (RDPN) confirmed by electromyography (EMG). All patients, who had not experienced satisfactory effects after glycemic control and neurotrophic drug treatment, experienced symptoms of RDPN, such as pain, numbness, and sensory changes in the limb extremities. The exclusion criteria were cervical radiculopathy, history of trauma, immune dysfunction, severe cardiopulmonary insufficiency, and obvious myasthenia.

This study was approved by the institutional review board of the Affiliated Hospital of Guizhou Medical University (Guiyang, China), and written informed consent was obtained from all participants.

**Figure 1:**
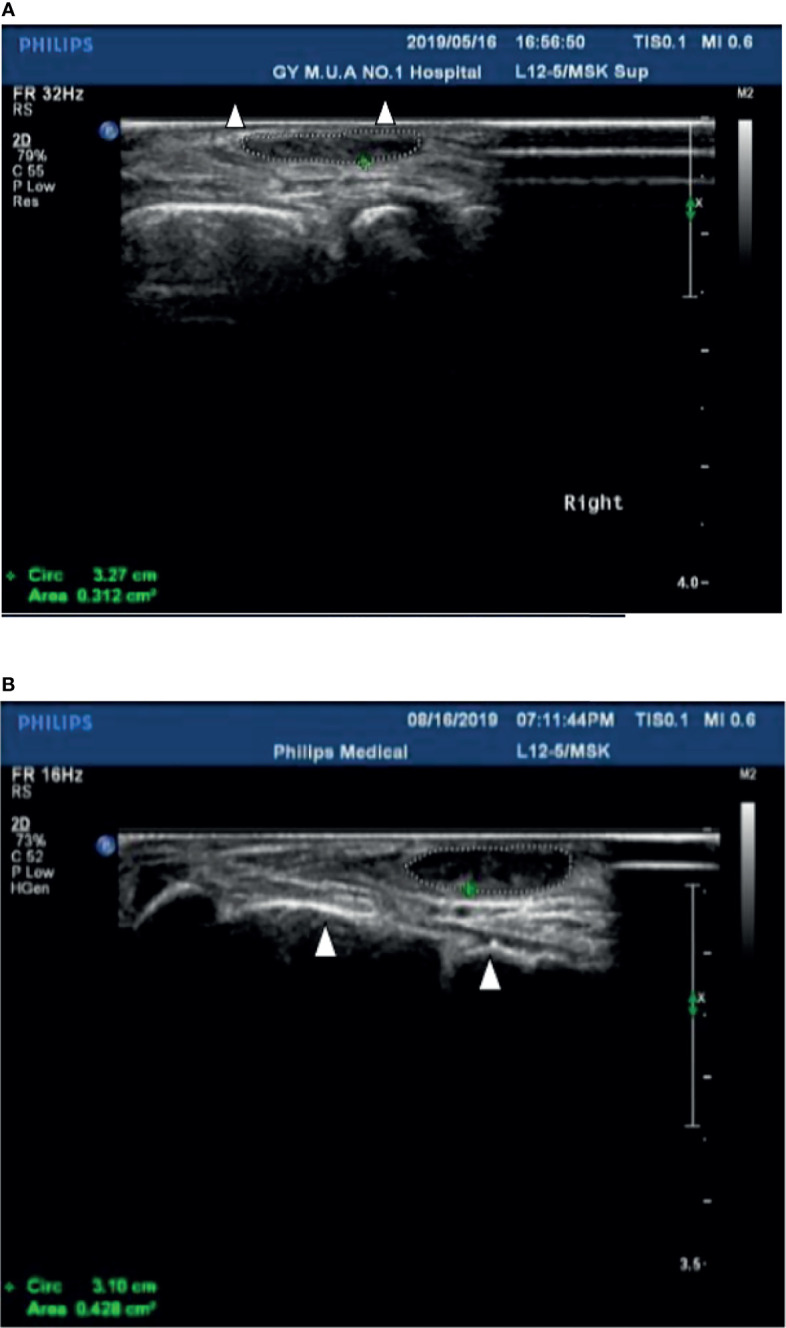
**(A)** The cross-sectional area of the extensor digitorum brevis of patient 1 before treatment was 0.312 cm^2^ (Circled by a dotted line); The Triangle refers to the fourth and fifth metatarsal bones. **(B)** The cross-sectional area of the extensor digitorum brevis increased to 0.428 cm^2^ (Circled by a dotted line) after two courses of treatment; The Triangle refers to the fourth and fifth metatarsal bones.

**Case 1**: A 60-year-old female with weak dorsiflexion of the feet and numbness for one year, occasionally with tingling, type 2 diabetes for five years, a maximum fasting blood glucose level of 15 mmol/L, and glycosylated hemoglobin of 6.9%. The diagnosis of DPN was based on numbness and tingling, slow conduction velocity (40.0 m/s) in the tibial nerves, and a waveform that vanished in the bilateral peroneal nerves as assessed by EMG examination. In the past year, the patient had experienced paresthesia and dyskinesia of the lower limbs. After treatment with insulin, her symptoms were somewhat relieved; subsequently, her glycemia was controlled with oral hypoglycemic drugs. The extensor digitorum brevis (EDB) muscle had significantly atrophied by 0.32 cm^2^ (**Figure 1A**), and the strength of the extensor of the thumb was grade 0/5 with steppage gait (**Table 1**).

**Table 1:** Clinical symptoms and signs of the five patients.

Patient	1	2	3	4	5
numbness	Numbness below the middle of the calf	Sock-like numbness in bilateral ankle	Persistent numbness below the middle of both calves	Persistent numbness below the knee joint	Persistent numbness below the knee joint
pain	Occasional tingling below the middle of the calf	Paroxysmal tingling, worse at night, affects sleep	none	none	none
weakness	Strenuous walking with others’ help, and occasionally falling	Bad mobility on lower limbs	Obvious weakness when squatting and standing	Bad mobility on lower limbs	Weakness after walking 100 meters
Clinical signs (both lower limbs)	Both sides of the back of the foot cannot be stretched back. The strength of the extensor of the thumb is 0,steppage and muscle atrophy, such as anterior tibialis, gastrocnemius, dorsi toe extensor, first and second interdigital	Bilateral foot analgesia, knee and ankle reflex (++), the strength of distal lower limbs are grade 5	Hypoalgesia below the middle of bilateral calves, persistent abnormal numbness, knee and ankle reflex (+), the strength of distal lower limbs is grade 5	Hypoalgesia, abnormal numbness below bilateral knee joints, tendon reflexes of lower limbs extremities, ankle reflexes (+), the strength of distal lower extremities is 5-level	Hypoalgesia, abnormal numbness below bilateral knee joints, tendon reflexes of lower limbs extremities, ankle reflexes (+), the strength of distal lower extremities is 4-level

After hospitalization, mecobalamin was started *via* ultrasound-guided injection of an appropriate dose (1 mg in 10 ml 0.9% NaCl). After therapy, the patient improved gradually. After one course of treatment, the foot strength returned to grade 2/5, with the calf circumference increasing from 25 to 28 cm (left) and 26 to 24 cm (right). The EMG results showed an insignificant change of 37.9 m/s in the bilateral peroneal nerve conduction velocity (**Table 2**). The cross-sectional area of the EDB was 0.43 cm^2^ after two courses of treatment (**Figure 1B**). The other changes are shown in **Tables 3** and **4**.

**Table 2:** Electromyogram examination results in patients before and after therapy.

Patients	1	2	3	4	5
pre-treatment	MNCV: Slowing conduction velocity(40.0m/s) on the tibial nerves and waveform vanished on bilateral peroneal nerves	SNCV: Without waveform on the left tibial nerve; EMG:Neurogenic damage on the left anterior tibial muscle.	MNCV: Tibial nerve: Left: 40.3m/s(34%↓), Peroneal nerve: Left: 38.6m/s(37%↓), SNCV: Without waveform on the tibial nerves.	MNCV: the peroneal nerves:Left:27.9m/s(52%↓); Right:36.2m/s; SNCV: without waveform on bilateral tibial nerves; Peroneal nerves: Left:60.5m/s,Right:77.1m/s	MNCV: Without waveform on bilateral common peroneal nerves; Tibial nerves: Left: 31.8m/s(24%↓); SNCV: Without waveform on bilateral common peroneal and tibial nerves.
post-treatment	MNCV: 37.9m/s on the bilateral peroneal nerves;Without waveform on bilateral peroneal nerves;	SNCV:Without waveform on the left tibial nerve.	/	/	MNCV: Without waveform on bilateral common peroneal nerves; SNCV: Without waveform on bilateral common peroneal and tibial nerves.

↓ indicates that the nerve conduction velocity is lower than the normal value.

**Table 3:** Evaluation of symptoms, ultrasound and cross-sectional area in patients.

		Patient 1	Patient 2	Patient 3	Patient 4	Patient 5
TCSS	pre-treatment	8	2	9	8	14
	post-treatment	5	7	6	6	11
DCEC	pre-treatment	4	7	6	7	7
	post-treatment	3	6	4	6	7
VAS	pre-treatment	2	5	0	6	4
	post-treatment	0	0	0	2	1
EBD	pre-treatment	0.32	1.24	1.34	1.19	1.2
	post-treatment	0.43	1.31	1.4	1.28	1.26
MIL	pre-treatment	1.86	2.71	2.95	2.27	2.43
	post-treatment	2.01	2.82	2.97	2.32	2.49

**Table 4:** Clinical manifestation changes after treatment.

Patient	Improvements after treatment				Adverse reactions
	Numbness	Painness	Strength	Remarks	
1	×	×	Muscle strength of extensor pollicis dorsalis of both feet increased from grade 0 to 2	The leg circumference (left) increased from 25cm to 28cm, (right) at 10cm below the patella of the leg, and increased from 24cm to 26cm (right)	Weakness increased after injection and improved after one hour.
2	×	×	The distal muscle strength of both lower limbs recovered from grade 5- to 5	The movement of lower limbs is more flexible than before, the running time of 5km is shortened from 50 min to 40 min, and the step distance is increased	Pain at injection sites and improved after a few minutes
3	×	×	The distal muscle strength of both lower limbs recovered from grade 5- to 5	When squatting and standing up, the movements of legs are obviously more flexible and vigorous than before	none
4	*	×	Distal muscle strength of both lower limbs grade 5	When walking, the step distance is larger than before	Weakness increased after injection and improved after 4 hours
5	*	×	The distal muscle strength of both lower limbs increased from grade 4- to 4+	One month after the follow-up treatment, the symptoms of numbness recovered to before the treatment	Local hemorrhage at injection sites occasionally

×Symptoms disappear; *Symptoms exist, but they are better than before.

**Case 2**: A 56-year-old male with type 2 diabetes for six years accompanied by renal function impairment. The diagnosis of DPN was based on characteristic sock-like numbness, inflexible movement of the lower limbs, no waveform in the tibial nerves, neurogenic damage to the left anterior tibial muscle upon electromyographic examination, and exclusion of other peripheral neuropathies. He had experienced paresthesia of the distal extremities within the previous two years. After treatment with mecobalamin and epalrestat based on strict control of blood glucose, these symptoms gradually worsened, with frequent nocturnal tingling affecting sleep. The foot strength was grade –5/5 with hypalgesia (**Table 1**).

After admission, mecobalamin was injected *via* ultrasound guidance at an appropriate dosage at the malleolus medialis of the posterior tibial nerves. Fortunately, the patient improved gradually after the therapy. After two courses, the foot strength returned to grade 5/5, the legs became more flexible, the walking distance increased, and there was no numbness or pain. An EMG examination showed no significant change in the bilateral peroneal nerves (**Table 2**). The other changes are shown in **Tables 3** and **4**.

**Case 3**: A 52-year-old male with type 2 diabetes mellitus for 11 years and retinopathy stage III diagnosed with DPN due to slow conduction velocity of the tibial and peroneal nerves and no tibial nerve waveform found with EMG. Mecobalamin and vitamin B1 were taken orally for nearly one year, but areflexia, distal numbness of the limbs, and weakness had occurred when squatting within the last six months (**Table 1**).

After two courses of treatment, the neurological examination showed that the muscle strength of the lower extremities was grade 5/5. Flexibility and numbness of the lower limbs improved, but no EMG results were obtained after treatment (**Table 2**). Moreover, after ultrasound-guided injection of saline and mecobalamin, the symptom and ultrasound image scores were improved (**Table 3**), and it was found that the EDB and muscles of the first interstitium (MIL) were larger than those before treatment (**Table 3**).

**Case 4**: A 76-year-old male with a history of type 2 diabetes for over 10 years diagnosed with DPN for 4 years. He had paresthesia and weakness in his lower limbs that was not relieved after treatment with mecobalamin, vitamin B1, and Maizhiling for more than two years. The EMG bilateral fibular motor nerve conduction velocity was 27.9 m/s (right, 52% ↓) and 36.2 m/s (left). The EMG showed that the conduction velocity of the bilateral tibial nerve waveforms had disappeared. The fibular sensory nerve values were 77.1 m/s (right) and 60.5 m/s (left) (**Table 2**). After one course of therapy, the patient’s walking distance increased, though with persistent numbness. The muscle strength of the lower limbs was not ameliorated at grade –5/5. The other relevant change scores, muscle cross-sectional areas, and thickness values are shown in **Table 3**.

**Case 5**: A 44-year-old male with type 2 diabetes for 13 years, renal failure and retinal complications, persistent numbness of the distal limbs, paresthesia, and weakness of the lower limbs after walking 100 m for nearly 5 years as a result of poor glycemic control, and 11.9% glycosylated hemoglobin. The patient had been treated with painkillers during this period. The waveform of the bilateral peroneal motor nerves had disappeared, and the conduction velocity of the tibial motor nerves was 31.8 m/s (24% ↓). Moreover, the waveforms of the bilateral tibial and peroneal sensory nerves were not revealed by EMG examination (**Table 2**).

After a course of treatment, the distal muscle strength of the lower limbs increased from grade –4/5 to +4/5, and the numbness was relieved gradually. Nevertheless, one month after follow-up, the symptoms appeared again. There was little change in the EMG results (**Table 2**). The other improvement indicators are shown in **Tables 3** and **4**.

### Therapeutic Procedure

**Figure 2:**
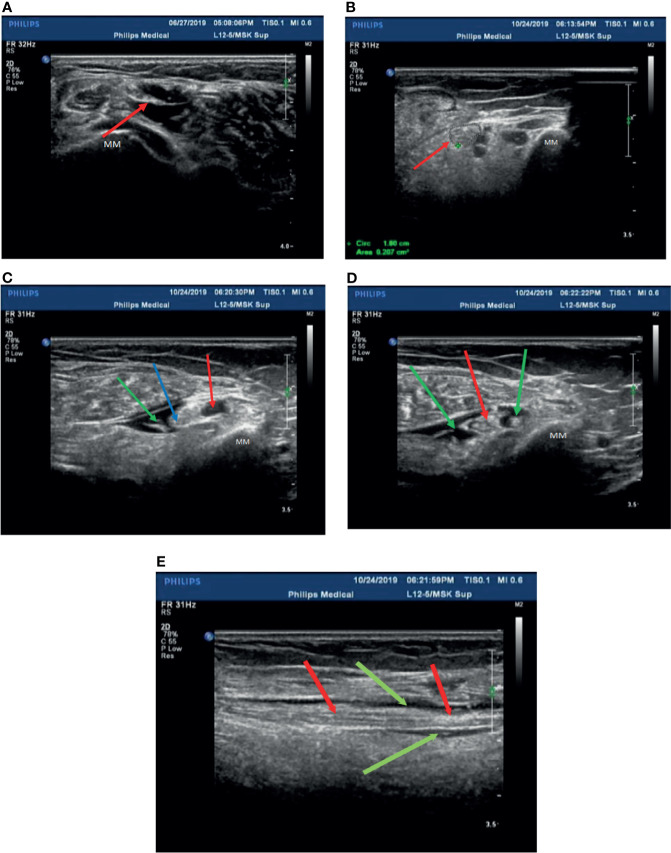
Patient 2 was treated with mecobalamin by ultrasound-guided hydrodissection injection at the malleolus medialis of the posterior tibial nerves **(A–E)**. **(A)** A normal posterior tibial nerve is shown as a clear neural boundary (cribriform network), normal internal echo (the posterior tibial nerve is below the red arrowhead). **(B)** The posterior tibial nerve of patient 2 was thickened with a fuzzy boundary in the medial malleolus (the red arrowhead indicates the posterior tibial nerve). **(C)** Injection with 3 ml saline at the medial malleolus of the posterior tibial nerve (the posterior tibial nerve at the red arrowhead, the point of a needle at the blue arrowhead, and the green arrowhead points to saline after injection). **(D)** The posterior tibial nerve was injected with mecobalamin at the medial malleolus after injection in the transverse section, and it can be seen that the solution is evenly wrapped around the posterior tibial nerve (red arrowhead pointed to the posterior tibial nerve, saline + mecobalamin at the green arrowhead). **(E)** The medial malleolus of the posterior tibial nerve after injection with mecobalamin in the longitudinal section, it can be seen that the fluid evenly covers the posterior tibial nerve (the red arrowhead points to the posterior tibial nerve, and saline + mecobalamin at the green arrowhead). MM; Medial malleolus.

In our experience, the fibular head of the tibial nerves, the section at the start of the peroneal nerves, and the medial malleolus of the posterior tibial nerves should be chosen as treatment injection sites after ensuring there are no wounds on local skin sites. Herein, these injection sites were sterilized with povidone iodine, and the ultrasonic probe was covered with gloves after having been sterilized. Two syringes were prepared before injection (first syringe: 10 ml of 0.9% sodium chloride injection; second syringe: 0.5 mg of mecobalamin injection + 9 ml of 0.9% sodium chloride injection to prepare a 10-ml mixed solution with 1.5–2 ml for each injection site). The posterior tibial nerve was displayed with a clear neural boundary, an internal echo, and a honeycomb-like structure by high-resolution ultrasound (**Figure 2A**). The puncture needle was as perpendicular to the skin as possible, entering along the puncture point at a distance about 0.5 cm from the center of the probe. About 45°–60° was required between the needle and sonographic probe (**Figure 2B**). Real-time observation of the position of the puncture needle tip during injection was performed when the needle was connected to the first syringe, and 3–5 ml of saline was injected around the nerve to separate the nerve water from the surrounding tissue by ultrasound-guided injection. At the end of the hydrodissection, a hypoechoic ring could be observed on the transverse axis (**Figure 2C**). Then, the needle tip was kept still and substituted with the second syringe to inject 1.5–2 ml of solution into the target nerve at each injection point (**Figure 2D**). The surrounding liquid around the nerve was shown clearly on the longitudinal axis (**Figure 2E**), after which the needle was removed.

It was essential to observe whether the patients had side effects in their limbs, such as numbness, weakness, and chills, during the injection process. Injection took place once every other day into the bilateral limbs, i.e., three times per week for four weeks per treatment course. Evaluation of the effect occurred after the treatment course. The injection operation was carried out by an experienced clinician and a sonographer.

### Assessment of Symptoms

The symptoms were evaluated with objectively measurable scores, including the VAS and Toronto Clinical Scoring System (TCSS).

The VAS is a scale to evaluate pain before and after therapy. The TCSS is a grading system for assessing the severity of DPN with a score from 1 to 19 (mild to severe) (11).

### Electrophysiological Examination

Each patient was diagnosed with DPN following an electrophysiological examination, wherein nerves were assessed with an electrophysiological severity scale comprising motor and sensory nerve conduction velocity detection. These tests were performed by a single physician using a Nicolet EDX EMG system (Madison, Wisconsin, U.S.A.). The measured nerves included the median nerve, ulnar nerve, tibial nerve, and common peroneal nerve. The sural nerve was not measured as it is easily affected by many factors. The motor conduction velocity of the median nerve and sensory conduction velocity of the ulnar and sural nerves, action potential amplitudes of all three nerves, and distal latency period of the median nerve in the wrist segment were determined.

The results were measured against the normal values obtained from the EMG room of Beijing Peking Union Medical College Hospital in China (12). Judging criteria: the examined nerve did not elicit potential (the statistical data were 0); the conduction velocity was over 20% lower than the normal low limit; the latency of the distal motor nerve exceeded 130% of the normal high limit; the amplitude of motor or sensory potential was decreased; the latency of the F wave exceeded 130% of the normal high limit; and the occurrence rate of the evoked wave was less than 50%.

Sensory nerve conduction measurement: The amplitude of the action potential of the sensory nerve decreases (particularly at the extremities of the lower limbs) although the conduction velocity is relatively normal, consistent with the characteristics of length-dependent axonal peripheral neuropathy. When there is compressive peripheral neuropathy, the sensory nerve conduction velocity at the embedded sites may be slowed. Among patients with autonomic nervous symptoms, sensory conduction can be normal. Thus, sensory nerve conduction measurement is helpful for the detection of subclinical lesions.

Measurement of motor nerve conduction: The latency and nerve conduction velocities of distal movement are usually normal in the early stage, and there is no partial conduction block of motor nerves or abnormal wave form dispersion. In later stages, the amplitude of the compound muscle action potential decreases, and the conduction velocity slows down slightly, similar to mononeuropathy or lumbosacral plexus disease. In patients with embedded peripheral neuropathy, the conduction velocity at the embedded sites can be significantly slowed.

### Sonographic Examinations

All nerve image examinations were performed using ultrasound guidance and evaluated by measuring the size of the morphologic changes of the nerves using an ultrasound system (Philips Ulrasound iU22, Holland) with a 5- to 12-MHz linear array transducer. The subjects lay on the examination bed and kept their lower limbs relaxed in the correct position according to different nerves. The cross-sectional areas of the tibial nerves were measured at the start of the nerves, the fibula head, and the medial malleolus of the posterior tibial nerves. Three averages of the cross-sectional area and three measurements for each site were used. The neuro-ultrasound scoring system included four aspects of the target nerves with ultrasound regarding the neuroimaging clarity of the cross-sectional area, internal echo, and the presence or absence of nerve entrapment (DCEC), of which the abbreviated form of the DCEC scoring system was used for each patient. The detailed scoring rules are shown in **Table 5** (13). Meanwhile, the extensor digitorum brevis (EDB) muscle and MIL were measured by ultrasound (14). The participants kept the ankle joint relaxed, and the cross-sectional area at the EDB midpoint and thickness of the MIL were determined.

**Table 5:** DCEC scoring rules DCEC scoring rules.

Clarity	Normal	0
	Under clear/slightly fuzzy	1
	vague	2
	Unclear	3
Cross-sectional area	Normal	0
	≤ 1.5 normal value range	1
	≥ 1.5 normal value range	2
Echoes	Normal	0
	Increase/decrease	1
Compression	N	0
	Y	1

DCEC scoring rules included clarity, cross-sectional area, echoes and compression. Clarity: ”normal” marked “0”; ”Under clear/slightly fuzzy” marked “1”; ”vague” marked “2”; ”unclear” marked “3”; Cross-sectional area: ”normal” marked “0”; ”≤ 1.5 times” marked “1”; ”≥ 1.5 times” marked “2”; Echoes: ”normal” marked “0”;”increase/decrease” marked “1”; Compression: ”N” means not compression, marked “0”; ”Y” means compression, marked “1”.

All patients were injected using ultrasound guidance. Before injection, the patients were examined for temperature, pinprick sensation (small fiber function), and vibration sensation using a 128-Hz tuning fork (for large fiber function). They were assessed with respect to neurophysiological changes, neural morphology, leg circumference, the EDB and MIL, and muscle strength in the bilateral limbs at the time of each injection.

## Results

The patients were evaluated at the pre-injection timepoint as well as after one or two injection courses. The mean age in this study was 57.6 ± 11.87 years, and the mean duration of the participants with DPN was 9.0 ± 3.39 years at the pre-injection baseline (**Table 6**). All five patients showed numbness and weakness in the lower limbs. A positive tendon reflex was found in three patients, and two patients had paroxysmal tingling pain in the lower limbs. However, only one patient had distal muscle atrophy of the lower extremities (**Table 1**). There were some slight adverse reactions after the injection, such as short-term limb weakness, bleeding, and pain at the injection site. However, these did not have serious consequences, and the patients recovered within hours.

**Table 6:** General characteristics and clinical signs of the patients (n = 5).

Patients	Sex	Age	Duration	Numbness	Pain	Weakness	Atrophy	Tendon reflex
			(year)					
1	female	60	5	+	+	+	+	–
2	male	56	6	+	+	+	–	–
3	male	52	11	+	–	+	–	+
4	male	76	10	+	+	+	–	+
5	male	44	13	+	+	+	–	–

Atrophy and Tendon reflex were examined for a positive (+) mark to check the inability of each specific test. If patients had these symptoms, numbness, painness and weakness, with positive (+) mark to express.

**Figure 3:**
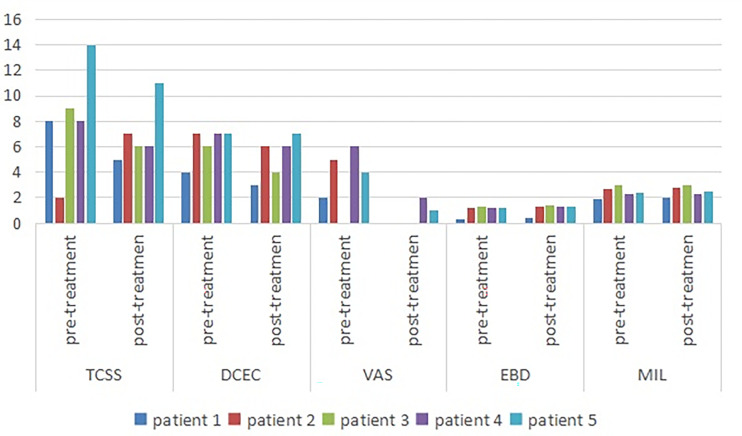
Symptom scores and morphologic changes were compared at baseline and post-injection. Among symptom scores, the VAS was significantly decreased, as compared to baseline. The DCEC score of nerves was measured by ultrasonography at limbs. Compared with baseline, the DCEC score and TCSS score showed a significant decrease post-injection. The EDB and MIL were increased after ultrasound-guided injection. DCEC, definition, cross sectional area, echogenicity and compression; TCSS, Toronto clinical scoring system; EDB, extensor digitorum brevis muscle; MIL, muscles of the first interstitium; VAS, visual analogy score.

Among the symptom scores, the VAS was decreased after the ultrasound-guided injection, with a mean of 3.4 points pre-injection and 0.6 points after treatment. The TCSS and DCEC scores did not significantly decrease after injection (**Table 3** and **Figure 3**).

The cross-sectional area of the EDB was measured by ultrasound at the midpoint of a straight line between the lateral malleolus and the fifth metatarsal tuberosity side of the dorsum pedis of the lower limbs, which determined the scanning plane. The cross-sectional areas of the EDB and MIL were decreased after injection (**Figure 1B**) compared with pre-injection, with a mean of 1.058 and 1.136 mm2, respectively (**Table 3**).

We observed that the electrophysiology outcomes did not significantly change compared with those pre-injection, though the conduction velocity from the left peroneal nerve increased slightly in one patient.

## Discussion

The purpose of this investigation was to explore the clinical value of ultrasound-guided hydrodissection in the treatment of RDPN. The results showed that the TCSS and VAS of patients with RDPN treated with ultrasound-guided injection improved to some extent, with the latter improving the most. There was insignificant change in the DCEC scores and neuroelectrophysiological results. The improved neurotrophic effect was reflected in the increase of the leg circumference, EDB muscle, and MIL. It was obvious for Case 1 that the distal small muscle of the lower extremity atrophied but then grew after two courses of treatment. This was detected by ultrasonographic examination and measurement of the EDB and MIL from the innervation of the fibular nerve and tibial nerve, respectively. The degree of foot muscle atrophy was helpful in evaluating the neuropathology and the treatment effect on peripheral nerves.

The possible therapeutic mechanisms of ultrasound-guided hydrodissection are as follows: (1) Use the cutting force of fluid instead of needle to separate soft tissue. The primary objective of hydrodissection is to release the entrapment of the peripheral nerves by hydrodissecting the nerves (15). (2) By separating adhesion, reduce nerve compression, improve peripheral vein and lymph reflux, so as to promote nerve function (16). In addition, local injection of mecobalamin around nerves promotes axonal growth, which has significant effects on sensory symptoms and short courses (17). Mecobalamin injection after hydrodissection distributes evenly among the surrounding tissues, increases absorption, and plays a more efficient role.

DPN is a type of compressive disorder. In DPN treatment, especially in patients with pain, numbness, and other symptoms, in addition to controlling glycemia, we found that surgical decompression had an obvious therapeutic effect. As a result of nerve thickening, it could easily be compressed by the narrow pipe. The decompression operation relieved the nerve entrapment in the patients, improved the blood supply of axoplasmic transport in peripheral nerves, and repaired injured nerves, which significantly reduced the pain symptoms compared with the former (18, 19). This research demonstrates the advantages of reducing nerve compression, improving the blood supply of nerves, increasing compliance, and reducing secondary nerve injury. The surgical decompression point was selected as the injection point of the hydrodissection in five patients. Mecobalamin is a type of endogenous co-enzyme B12 and can easily be transferred to the organelles of nerve cells in the process of methionine synthesis from homocysteine. It can promote the synthesis of nucleic acids and proteins, the transport and regeneration of axons, and the synthesis of phosphatidylcholine in myelination, whereby it has obvious effects on damaged nerve cells (20). Li Yuxin et al. carried out a study of axillary brachial plexus block compared with the traditional perivascular injection method and identified that expansion of the peripheral nerve space by ultrasound-guided saline reduced anesthetic doses (21). Consistent with our findings, earlier studies by Ide et al. also described that intrathecal injection with mecobalamin improved symptoms in patients with RDPN; however, the observation time was short, and nerve repair was not detected by the electrophysiological examination, which may explain why the EMG outcomes, including autonomic neural function, were insignificantly improved (22).

### Limitations

In this study, only five patients were observed for a short time, and a standard could not be formed. In this study, patients with refractory diabetic peripheral neuropathy have been treated with different hypoglycemic and vegetative nerve regimens. The severity of the lesions may be different before using ultrasound-guided hydrodissection. In addition, the objective score of patients with symptoms is relatively low, and the response to treatment may not be comprehensive. However, as a small case report, this study is reasonable from the comparison before and after treatment and the selection of research objects. Otherwise, without treatment with hydrodissection and local mecobalamin injection, we cannot combine the results with the efficacy of hydraulic exfoliation, local injection of mecobalamin combined with efficient nutrition, or both. Thus, a large number of participants and researchers will not be able to distinguish the results from the efficacy of hydrodissection, local injection of mecobalamin, or a combination of both. In the future, we will recruit more participants to further study the treatment and prognosis, and we need to design a large sample size, controlled and multicenter study to determine whether ultrasound-guided hydrodissection has satisfactory efficacy in patients with RDPN compared with traditional methods.

## Conclusion

In our patients, the nerves were thickened and obviously damaged at compression sites, and the recovery time was measured by electrophysiology improvements. Owing to the advantages of nutrition and safety in relieving nerve compression, the pain of the patients with RDPN was relieved. Therefore, we believe that the most effective method for the treatment of DPN is to reduce nerve injury by controlling blood sugar.

## Data Availability Statement

The original contributions presented in the study are included in the article/supplementary material. Further inquiries can be directed to the corresponding author.

## Ethics Statement

The studies involving human participants were reviewed and approved by Ethics Committee of Guizhou Medical University. The patients/participants provided their written informed consent to participate in this study. All participants provided written informed consent for the publication of the case reports (including all data and images).

## Author Contributions

HQH and SW conceived the idea and conceptualized the study.HLH, CT, ML and JCL completed the ultrasound for patients. JH performed nerve conduction tests. HQH completed the process of hydrodissection injection.HLH and HQH collected and analyzed the datas. Finally, HLH drafted and reviesd the manuscript,then YL and SW reviewed the manuscript.All authors contributed to the article and approved the submitted version.

## Funding

Application and industrialization project of scientific and technological achievements in Guizhou Province, “The regional pilot of precision medical poverty alleviation powered by technology— Sub Project III, diagnosis and treatment of epilepsy, Parkinson’s disease and headache”, [Achievements of science and technology synthesis in Guizhou (2018) 4615-3]; National Key R&D Program of China (NO.2018YFC1312901); Guiyang Science and Technology Program, “the establishment and clinical application of the neuroultrasound scoring system”, [architecture contracts (2019) 9-1-7].

## Conflict of Interest

The authors declare that the research was conducted in the absence of any commercial or financial relationships that could be construed as a potential conflict of interest.

## Publisher’s Note

All claims expressed in this article are solely those of the authors and do not necessarily represent those of their affiliated organizations, or those of the publisher, the editors and the reviewers. Any product that may be evaluated in this article, or claim that may be made by its manufacturer, is not guaranteed or endorsed by the publisher.
